# Supporting People Who Have Lost a Close Person by Bereavement or Separation: Protocol of a Randomized Controlled Trial Comparing Two French-Language Internet-Based Interventions

**DOI:** 10.2196/39026

**Published:** 2022-06-23

**Authors:** Anik Debrot, Maya Kheyar, Liliane Efinger, Laurent Berthoud, Valentino Pomini

**Affiliations:** 1 Cognitive and Affective Regulation Laboratory Institute of Psychology University of Lausanne Lausanne Switzerland

**Keywords:** internet-based interventions, grief, bereavement, separation, divorce, identity, digital health, mental health, psychotherapy

## Abstract

**Background:**

Internet-based interventions (IBIs) are as efficient as face-to-face psychotherapy for a variety of mental health disorders, including complicated grief. Most evidence stems from guided IBIs. However, recent research indicates that the benefit of guidance is lower in more interactive IBIs. As such, providing guidance only to people requiring it (guidance on demand) appears a cost-effective solution. This is particularly important to develop given the recent rise in grief symptoms in the context of the COVID-19 pandemic. This paper presents the protocol of a randomized controlled trial comparing the efficacy and adherence rate of 2 IBIs for grief-related symptoms after the loss a close one following death or romantic separation, using a guidance on demand framework. LIVIA 2.0 was developed based on theoretical and empirical findings on grief processes and IBIs, and it will be compared to LIVIA 1 that has already demonstrated its efficacy.

**Objective:**

Our main hypotheses are that LIVIA 1 (control condition) and LIVIA 2.0 (experimental condition) increase participants’ well-being and decrease their distress at posttest and at follow-up, that LIVIA 2.0 is more efficient than LIVIA 1 for all outcomes, and that LIVIA 2.0 has less dropouts than LIVIA 1.

**Methods:**

Outcomes will be assessed at pretest, posttest (12 weeks later), and follow-up (24 weeks later). We will recruit 234 participants through a variety of means, including social media and contacts with the press. Primary outcomes are grief symptoms, depressive symptoms, and eudemonic well-being. Secondary outcomes are anxiety symptoms, grief coping strategies, aspects related to self-identity reorganization, and program satisfaction. LIVIA 2.0 participants will additionally undergo a weekly mood and grief symptom monitoring, allowing us to explore the short-term efficacy of the sessions.

**Results:**

The creation and development of the content of LIVIA 2.0 was completed during the first phase of the project. Participant recruitment will begin in May 2022 and will last until January 2023.

**Conclusions:**

This study will emphasize the relevance of the innovations included in LIVIA 2.0 regarding the efficacy and dropout rate of IBIs for grief symptoms and will allow investigations on how these changes impact the demand for guidance. In the current postpandemic times, developing and assessing IBIs targeting grief symptoms are particularly critical given the rise in grief-related symptoms.

**Trial Registration:**

clinicaltrials.gov NCT05219760; https://tinyurl.com/3dzztjts

**International Registered Report Identifier (IRRID):**

PRR1-10.2196/39026

## Introduction

### Background

Internet-based interventions (IBIs) offer numerous efficient prevention and treatment programs for a variety of psychological difficulties [1,2]. Generally based on methods originating from empirically supported face-to-face psychological interventions for people reporting complicated grief symptoms [3,4], IBIs for grief-related symptoms are also effective [5,6].

Offering guidance to participants is one of the most commonly cited means to improve IBI effectiveness [7], including grief-related symptoms [5]. However, recent evidence indicates that the benefits of guidance are lower in more interactive internet interventions [8]. Moreover, when given the choice, not all participants request guidance. Additionally, the efficacy of guidance on demand is similar to standard weekly guidance [9-11]. Finally, including specific and individualized principles, such as the Motive-Oriented Therapeutic Relationship (MOTR), in the guidance appears feasible and useful for IBIs [12,13]. Thus, MOTR-based guidance on demand appears as a cost-effective alternative to mandatory guidance. Other elements can also improve adherence rate and efficacy: (1) using automated reminders [14]; (2) providing interactivity in the tasks and exercises [15-18]; (3) promoting personal resources to cope when problematic experiences arise [19,20]; (4) tailoring the intervention to the participant’s characteristics and timing the content in accordance with the participant’s characteristics [21-24].

LIVIA 2.0 was developed as an alternative IBI to LIVIA 1, the original program, for grief-related symptoms after bereavement or separation [25], which was tested in German via a randomized controlled trial (RCT) [26] and in French via a noncontrolled trial [27]. Indeed, fundamental research has pointed to the many similarities between these kinds of losses [27,28]; moreover, studies on LIVIA 1 have proved that the same intervention can be provided to both populations [26,29]. LIVIA 2.0 implements different factors to improve patient adherence and program efficacy. Specifically, it includes automated emails, increased interactivity (quizzes, video files, and audio files), personal resource assessment and promotion, and the freedom to choose the session order combined with an individualized recommendation. The content is based on the Dual Process Model (DPM) of bereavement recovery and proposes an oscillation process mimicry [30,31] with an alternation of loss-focused and restauration-focused sessions. Finally, LIVIA 2.0 includes a module focused on identity and memory processes that play a key role in adapting to loss [32,33] as well as novel emotion regulation tools [34,35].

In detail, the development of LIVIA 2.0 and its innovations were based on the theoretical and empirical literature about grief and romantic dissolution. On the theoretical level, we relied on one of the most influential models of coping with loss, the DPM of Coping with Bereavement [30,31]. According to it, after loss, instead of going through consecutive phases, people oscillate between a focus on the loss and a focus on the restauration from the loss. This model postulates that oscillation is a natural and necessary movement to cope with loss. Moreover, DPM-based interventions are more efficient than classic ones [36]. Hence, LIVIA 2.0 imitates the oscillation process by alternating between loss- and restauration-focused sessions. Additionally, LIVIA 2.0 integrates recent loss-related empirical findings into its content and exercises. For example, the Emotion module proposes self-compassion exercises, as self-compassion has been shown to predict better grief recovery [37,38]. Moreover, LIVIA 2.0 includes a newly developed module based on empirical cognitive psychopathological knowledge and focused on identity processes, which play a key role in adaption to bereavement [32,33]. Addressing identity factors, such as fostering an independent sense of identity by focusing on adaptive specific autobiographical memories and future projections, could improve existing cognitive-behavioral programs for grief.

LIVIA 2.0 also integrates recent developments in IBIs [16,20]. First, a series of changes are designed to improve participant autonomy by sending automated emails [14], providing individualized recommendations about the order in which to complete the modules [22], promoting and encouraging the use of personal resources [20], and augmenting the interactivity of the website [15-18]. Finally, the emails exchanged with the participants within the guidance on demand framework will be based on the MOTR [12].

### Objectives

Our main hypotheses are the following: (1) LIVIA 1 and LIVIA 2.0 increase participants’ well-being and decrease their distress at posttest and follow-up. (2) LIVIA 2.0 is more efficient than LIVIA 1 for all outcomes. (3) LIVIA 2.0 has less dropouts than LIVIA 1.

Moreover, we will conduct the following exploratory analyses. First, we will compare the guidance requirements of the participants (ie, number of participants requiring guidance and number of exchanged emails) in LIVIA 2.0 with those of the LIVIA 1 participants and explore which session triggers more requests for guidance. Second, we will examine in LIVIA 2.0 the short-term effectiveness of each module on the participants' weekly moods, feelings of loneliness, and grief symptoms. Third, we will compare participant satisfaction in the 2 versions of LIVIA. Fourth, we will explore the role of multiple measures (attachment style, type of loss, interpersonal closeness to the lost person, and symptom severity) as moderators of the program’s effectiveness. Finally, we will investigate the semantic content of the responses to the LIVIA 2.0 exercises [39] to explore its relationship with improvement over the evaluation period.

## Methods

### Study Setting

This is a study of an IBI in French. We will recruit participants in Switzerland, but participation will be open to French-speaking people across the world.

### Design and Procedure

**Figure 1:**
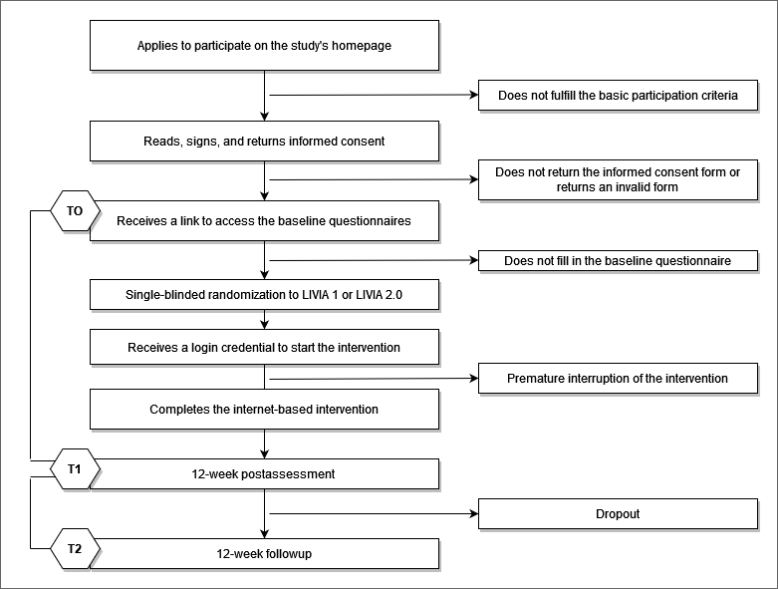
Flowchart of the study design. T0: assessment at baseline; T1: assessment at posttest; T2: assessment at follow-up.

This study is a monocentric, single-blinded, 2-arm RCT comparing the efficacy of 2 versions of an IBI—namely LIVIA 1 and LIVIA 2.0—aimed at relieving distress and augmenting the well-being of people suffering from prolonged grief symptoms. There will be three measurement points: a pretest (T0), posttest (T1), and follow-up (T2). The flowchart of the study design is presented in Figure 1.

This self-help intervention is embedded in a larger project on life-span vulnerabilities and strengths conducted by the LIVES Centre [40].

### Study Conditions

#### LIVIA 1: Control Condition

LIVIA 1 is a self-help intervention for coping with prolonged grief symptoms after the death of a loved one or romantic separation/divorce, developed at the University of Bern by Brodbeck and colleagues [25]. Participants are encouraged to work through 1 session per week and complete the exercises provided. Each session takes approximately 60 minutes to complete. The sessions must be completed in the prescribed order. Details of the session contents can be found elsewhere [25].

#### LIVIA 2.0: Experimental Condition

LIVIA 2.0 is a psychological IBI composed of 10 sessions (see Table 1). These include an introductory session, a closing session, and 8 sessions in between belonging to 4 modules. Theoretically anchored in the DPM [30,31], each module comprises a session focused on loss and another on restoration. The modules have the following main themes: cognitions, emotions, behaviors, and identity. The sessions involving these modules are composed of psychoeducational information and exercises. In each session, participants can choose between 3 variants of the exercise (eg, some exercises vary in their themes or length). They are expected to complete 1 exercise per session but can complete all 3 available exercises if they wish to. LIVIA 2.0 also contains texts, audio and video files, and interactive quizzes.

**Table 1:** Summary of the sessions and main content of LIVIA 2.0^a^.

Session	Module	Theme	Content
1	Introduction	Psychoeducation; assessment of resources and goals	Information about the self-help intervention, grief reactions, predictors, and treatment of complicated grief; assessment of individual resources and goals in pursuing the intervention
2	Cognition-focused	Loss-oriented session	Information about the impact of negative thoughts on well-being and the typical negative thoughts experienced during difficult grief; cognitive restructuration exercises
3	Cognition-focused	Restoration-oriented session	Information about secondary stressors and related thoughts; importance of building positive thoughts as resources; exercise to promote focus on positive aspects of one’s own life
4	Emotion-focused	Loss-oriented session	Information about the central role of emotions in the grieving process; assessment of own emotional state; self-compassion exercises
5	Emotion-focused	Restoration-oriented session	Importance of experiencing positive emotions, even if only briefly; hypnosis-like exercises to promote positive emotions
6	Behavior-focused	Loss-oriented session	Information about the typical vicious circle of avoidance in grief and the importance of confronting the avoided situations; confrontation exercises
7	Behavior-focused	Restoration-oriented session	Importance of behavioral activation in line with one’s own values; assessment of values; preparation of behavioral activation in line with one’s own values
8	Identity-focused	Loss-oriented session	Psychoeducation about identity formation and the way it is affected by grief; exercise aimed at revisiting memories and the relationship with the lost person; developing an independent sense of identity
9	Identity-focused	Restoration-oriented session	Psychoeducation about the importance of autobiographical memory for the individual's sense of self and ability to generate images of future events; exercise aimed at focusing on specific adaptive autobiographical memories and future projections to foster an independent sense of identity
10	Conclusion	Assessment of the individual’s experience of the intervention; relapse prevention	Promoting reflection on one’s own journey through the program (what was learned, what still needs to be done); identification of vulnerable moments and strategies to deal with the latter

^a^Modules 2 to 9 can be completed in the order chosen by the participants.

#### Navigation in the LIVIA 2.0 Program

The participants will first complete the introduction module. In this module, they will answer a questionnaire for allowing the program to automatically suggest an order of completion for the main modules (sessions 2 to 9, see Table 1), based on each participant’s priorities (eg, “It is important for me to avoid being taken over by negative thoughts” or “It is important for me to experience more positive emotions”). Nevertheless, participants will be able to choose the completion order freely. Each module consists of a (1) short introduction, (2) loss-related session, and (3) restauration-related session. Participants will mandatorily complete the module in this order. Once they have completed the 4 modules, they will complete the program with the conclusion session.

The following are the rules governing content access: (1) Once a session is opened, only that session is available for the next 7 days. After that, the following session is available, or if starting a new module, participants can choose the next module they wish to work on. (2) Within a session, participants can only access the content of that session or content that has already been completed. (3) Once they have reached the end of a session within a module, they can choose to do another exercise. (4) At the end of the intervention (12 weeks after starting it), they will have access to all the content.

#### Additional Resources

To assist participants as they complete the program, a toolbox will be provided with the content shown in Textbox 1.

**Toolbox provided to the participants during the program.**

**Favorites**

Participants will be able to add parts of the program that they particularly appreciate to their favorites.

**Individual resources of the participant**

Participants can access the assessment of their personal resources with AERES (a self-assessment scale for resources) during the first session (Bellier-Teichmann et al [20]).

**Soothing techniques for emotionally difficult times**

Short exercises, such as breathing exercises or tools to anchor attention in the present moment, will be available.

**Key scientific references**

We will provide the main references of the content of each session so that participants may deepen their knowledge on a specific domain, should they wish to.

**Automated emails**

To encourage participation, emails will automatically be sent (1) when a new session becomes available and (2) if the participant has not accessed the program for 7 consecutive days.

### Assignment of Interventions

To assign participants to the LIVIA 2.0 (experimental) or LIVIA 1 (control) condition, we will use the randomization module in REDCap (Research Electronic Data Capture [41,42]), which will automatically generate a 1:1 randomization. We will apply a single-blinded randomization strategy stratified according to the gender and loss type (bereavement vs separation) of the participants. Due to the nature of the study, double-blinded randomization is not possible.

### Participants

#### Sample Size

We will conduct a power analysis with G*Power (Version 3.1.9.2 [43]) for ANOVA of repeated measures involving within-between interactions that would enable us to compare the efficacy of the 2 interventions. Based on a probability level of 0.05 and a power of 0.8, to detect a small effect size of *f* =0.1 (as we expect small differences between the 2 programs), a sample size of 164 participants is needed. Expecting a dropout rate of 30% at the 3-month follow-up, we aim to have 234 participants at posttest.

#### Recruitment

To maximize recruitment, the following strategies will be applied: (1) contacting grief and divorce-related associations as well as other potentially interested associations (eg, senior citizens’ associations), (2) contacting radio and television channels as well as newspapers, (3) distributing flyers in churches, beauty salons, and health-related institutions, (4) promoting the project in church administrations, (5) sending emails to large groups of university students, (6) promoting the study through social media, and (7) publishing an advert on research facility websites. To ensure an ongoing flow of participants until we reach the desired number of participants, we will continuously revise and rerun our recruitment strategy.

#### Eligibility Criteria

Participants meeting all the following inclusion criteria will be eligible for the study: (1) have experienced bereavement or separation, (2) either events must have happened more than 6 months prior to participating in the study, (3) feel the need for support to cope with the loss (a diagnosis of complicated grief is not necessary), (4) aged 18 years or older, (5) have regular access to the internet, (6) fluent in French, and (7) have approved the informed consent form.

The presence of any of the following exclusion criteria will preclude individuals from participating in the study: (1) moderate to acute current suicidality (Suicidal Ideation Attributes Scale score>19] [44,45]) (Note that participants having a Suicidal Ideation Attributes Scale score between 13 and 19 will undergo a telephonic interview to accurately assess their suicidal risk and will be excluded from the study if the risk is assessed as important [46]), (2) severe psychological or somatic disorders that need immediate treatment, (3) concomitant psychotherapy, (4) prescription or change in dosage of psychoactive drugs in the month prior to or during the self-help intervention, (5) inability to follow the study procedures (eg, due to comprehension problems), and (6) enrolment of the investigator, their family members, employees, and other dependent people.

### Security During the Procedure

During the intervention, participants will receive a biweekly assessment of the occurrence of a serious adverse event. Additionally, they will be able to contact the investigation team at any time through a contact form available in both the IBIs. An automatic reply will be sent informing that their message will be processed within 3 working days and that they can contact the emergency numbers that will be provided. This will enable the participant to make contact and be attended to at any moment if necessary. We will then discuss with them the reason for contacting us and if a serious adverse event is occurring. If this is the case, the participants will be contacted, and their situation will be evaluated. If necessary, alternative treatments will be proposed.

### Measures

The calendar for obtaining the measures from the participants can be found in Multimedia Appendix 1.

#### Primary Outcome Measures

Grief symptoms will be assessed with the French version of The Traumatic Grief Inventory-Self-Report prepared by Cherblanc and Zech (unpublished work, 2021), an 18-item self-report measure assessing the presence of symptoms described in each item on a 5-point scale ranging from 1 (never) to 5 (always) [47]. This inventory is designed to assess symptoms of persistent complex bereavement disorder as described in the Diagnostic and Statistical Manual of Mental Disorders (5th edition [48]) and prolonged grief disorder in the International Classification of Diseases (11th edition) [49]. It has shown good reliability and validity to recognize people at risk of prolonged grief disorder.

Depression symptoms will be assessed with the Patient Health Questionnaire-9 [50], a 9-item measure of depression with adequate reliability and validity [51,52]. It assesses various depressive symptoms in the previous 2 weeks on a scale ranging from 0 (never) to 3 (almost every day).

Well-being will be measured with the French version [53] of the Flourishing Scale [54], a brief 8-item summary measure of the respondent’s self-perceived success in important areas such as relationships, self-esteem, purpose, and optimism. As such, it measures eudemonic well-being, a larger conception of conventional well-being measures. Participants will answer questions such as “I lead a purposeful and meaningful life” on a scale ranging from 1 (strongly disagree) to 7 (strongly agree).

#### Secondary Outcome Measures

Anxiety symptoms will be assessed with the Generalized Anxiety Scale [55] in its validated French version [56]. The scale has 7 items (eg, “Feeling nervous, anxious, or on edge”) assessing the frequency of symptoms over the previous 2 weeks rated on a 4-point Likert scale (0=not at all; 3=nearly every day).

Grief coping strategies will be measured with the Coping with Bereavement Questionnaire [57]. It consists of 14 items relating to loss orientation, such as “I take time to think about the things that I have experienced with the lost person” and 12 items relating to restoration orientation, such as “I try to accept living on without the lost person.” These items correspond to a list of coping strategies (thoughts, behaviors) for which the respondent must estimate the frequency of use during the previous month (1=almost never [less than once a month]; 5=all the time [several times a day]; 0=not applicable [this statement does not apply to the context of my life]).

Aspects related to identity will be measured with 3 scales. First, the 12-item French version [58] of the Self-Concept Clarity Scale [59] will assess the extent to which self-beliefs are clearly and confidently defined, internally consistent, and stable. Participants will answer questions such as “In general, I have a clear sense of who I am and what I am,” rated on a 5-point scale (1=strongly disagree to 5=strongly agree). Second, the Centrality of Event Scale [60] will assess the extent to which a memory for a distressing life event becomes a reference point for personal identity and for the attribution of meaning to other experiences in the person's life (eg, ”I feel that this event has become a central part of my life story.”), rated on 5-point scale (1=totally disagree to 5=totally agree). The French version of the Centrality of Event Scale was developed by Ceschi et al (unpublished work). Finally, 3 items will assess self-continuity [61]: “I am the same person as I always was,” “With time a lot of things have changed, but I’m still the same person,” and “I am a different person than I was in the past.” These items will be evaluated on a 5-point scale (ranging from 1=does not apply to me at all to 5=fully applies to me).

The feelings of loneliness will be measured with the University of California Los Angeles Loneliness Scale [62,63] contains 10 positive (eg, “I feel in tune with the people around me”) and 10 negative (eg, “I lack companionship”) items. Participants will respond to each item using a 4-point scale (1=never to 4=often).

Program satisfaction will be measured with a translated version of the Client Satisfaction Questionnaire adapted to IBIs [64]. The questionnaire will assess satisfaction with the theoretical content, practical content (exercises), structure, design, and overall assessment of the intervention. It contains 15 items rated with a scale ranging from 1 (no) to 4 (yes) and 4 open-ended questions.

Additionally, we will monitor the mood of the LIVIA 2.0 participants on a weekly basis using a single item: “How would you describe your current mood” on a scale ranging from 0 (very bad*)* to 6 (very good). Moreover, we will monitor weekly grief and solitude symptoms on a scale ranging from 0 (strongly disagree) to 7 (strongly agree) with the following items: “During the past 24 hours, (1) I have felt negative emotions; (2) I have had negative images or thoughts; (3) I felt blocked in my behavior (what I do, my activities); (4) I felt lonely; (5) I had a very clear vision of myself.” We will also assess the linguistic behaviors in the exercises of LIVIA 2.0 where the participants are required to describe a loss-related situation. More specifically, we will analyze indicators of verbal immediacy (use of first-person pronouns and present-tense words) and so-called “we-talk” (first-person plural pronouns [39]). Finally, we will assess the degree of guidance required in each group (number of participants requiring guidance and number of emails exchanged).

#### Predictors and Moderators

The following variables will be assessed only at baseline (T0) and their moderating role in the efficacy of the intervention will be explored: (1) demographic characteristics (31 items); (2) relationship quality prior to death or separation, measured with adapted items from the Dyadic Adjustment Scale-4 [65,66]. The Dyadic Adjustment Scale-4 will only be used if the person lost was a romantic partner. It has 4 items, with 3 items (eg, “In general, how often do you think that things between you and your partner are going well?”) rated on a 6-point scale (from 0=all the time to 5=never) and a 4th item rated on a 7-point scale (from 0=extremely unhappy to 6=perfectly happy); (3) adult attachment style, measured with the Experiences in Close Relationships–Short Form scales [67,68]. This 12-item measure captures variability along two attachment dimensions: avoidance and anxiety. Participants will rate the extent to which they agreed with each statement using a 7-point scale (1=strongly disagree to 7=strongly agree); (4) interpersonal closeness with the person lost measured with the Inclusion of Other in the Self scale [69]. Respondents will be required to select 1 out of 7 Venn-like diagrams depicting their relationship with the lost person.

### Data Collection and Management

Study data will be collected and managed using REDCap electronic data capture tools hosted at the Lausanne University Hospital [41,42]. REDCap is a secure, web-based software platform designed to support data capture for research studies, providing (1) an intuitive interface for validated data assessment, (2) audit trails for tracking data manipulation and export procedures, (3) automated export procedures for data downloads to common statistical packages, and (4) procedures for data integration and interoperability with external sources. Data integrity is enforced through several mechanism (ie, referential data rules, valid values, range checks, and consistency checks).

Moreover, data on the use of the self-help sessions will be collected within the platform, as well as the entries of the participants in LIVIA 2.0 (exercises, quizzes, questions, etc). All data will be saved anonymously, identified only by a random code. The servers are protected by high-end firewall systems. The participant code list will be saved on an internal Network-Attached Storage. Only the researchers directly involved in the study will have access to the data.

### Statistical Analysis

Primary analyses will be performed using SPSS (IBM Corporation) and R (R Foundation for Statistical Computing) after the participants complete the study. Primary follow-up analyses will be conducted 3 months later. Intermediate analyses during data collection will be done. We will perform intention-to-treat analyses [70]. We will develop multilevel mixed effects models with repeated measures data in SPSS to evaluate the efficacy of LIVIA 2.0 compared to LIVIA 1 and the stability of the effects. These models have the advantages of considering the dependency of the data and accounting for the correlation of repeated measures within individuals. Moreover, they rely on all available data of every participant and estimate parameters of missing values [71].

We will explore the potential moderating effect of different variables on the intervention effects by conducting analyses of covariance for repeated measures. To explore the short-term efficacy of each module, we will use multilevel modeling whereby each participant’s monitoring data are nested within participants. These analyses will be conducted with Mplus [72] or R. Additionally, we will analyze qualitatively the satisfaction questionnaire using the thematic content analysis method [73]. Finally, we will analyze the semantic content of the exercises included in LIVIA 2.0 [39] and test if some categories (eg, “we-talk”) are associated with the efficacy of the program. We will rely on a significance level of a 2-sided *α*=.05 or smaller. We will use the Bonferroni correction to adjust for multiple testing.

To handle missing data and dropouts, we will conduct the analyses relying on the intention-to-treat paradigm. We will first analyze the magnitude of missing data, explore the missing data patterns, and determine the pattern of missing data (missing completely at random, missing at random, and not missing at random). If the missing mechanism is missing at random, we will use multilevel regression analyses, which allow for nonindependent observations and for different numbers of measurement points per participant and are thus less sensitive to missing data [71].

### Monitoring of the Study

The Clinical Trial Unit of the Centre de Recherche Clinique (CTU/CRC) of the Lausanne University Hospital will monitor the study. This includes (1) a monitoring preparation meeting; (2) an initiation visit whereby before starting the study, the CRC team and the investigation team will go over the entire procedure of the clinical trial; (3) intermediary visits whereby the CTU/CRC will control the available data and write reports on a regular basis; and (4) a close-out visit whereby upon study completion, the CRC team and the investigation team will meet 1 last time and revise all the study materials.

### Ethics Approval and Consent to Participate

The protocol (trial registration: NCT05219760) has been approved by a federally acknowledged ethics committee (Commission cantonale d'éthique de la recherche sur l'être humain, CER-VD, BASEC reference number: 2021-D0086) and by the Swiss Agency for Therapeutic Products (reference number: 102667545) in accordance with the Swiss Ordinance 810.306 on Clinical Trials with Medical Devices. We will obtain informed consent from all participants in the study (see Multimedia Appendix 2), codify their data, and ensure secure storage of the data.

## Results

The project started in February 2019. Throughout the initial years of the project, the website and the content of the LIVIA 2.0 intervention were developed, pretested [74], and corrected according to the obtained feedback. Additionally, the LIVIA 1 intervention was transferred to a new digital platform and the study materials were selected and prepared. Moreover, the required approvals by the ethics committee and the competent authority (Swissmedic) were prepared and obtained. At the time of the submission (April 2022), the website and research materials were ready, but no data had been collected. The recruitment will begin in May 2022, once the monitoring authority provides approval and will last until January 2023 (the recruitment period might be prolonged if necessary). The findings will be disseminated using different methods, including peer-reviewed journals, academic and public conferences, and other verbal and digital channels (eg, through the blog tab of the study’s webpage or using the social media accounts of the study).

## Discussion

In this study, we aim to investigate how a newly developed IBI for grief-related symptoms (LIVIA 2.0) compares to one that has already been tested and validated (LIVIA 1 [26,29]) for people who have lost a close one either by bereavement or separation. This will inform about the efficiency of incorporating different empirically based innovations.

### Strengths and Limitations

The treatment gap in mental health care is a global reality [75], and we can expect the COVID-19 crisis to have aggravated it [76]. Moreover, because of the increased deaths and obstacles to social contact due to the distancing measures during the pandemic, it is particularly crucial to make IBIs available for general purposes [77] and for grief symptoms in particular [78]. To our knowledge, this study will be the first one to test an IBI targeting grief symptoms in French. As such, it will contribute toward facilitating access to high-quality IBIs for the French-speaking population. Additionally, research on IBIs using a guidance on demand framework is rare [10,11]. Hence, this study will contribute to knowledge about this method that may provide a promising outlook from human resource and economical perspectives. This project will thus contribute toward expanding the possibilities to offer accessible and cost-efficient interventions to people in need.

Some potential limitations can be anticipated. First, the self-selection of the sample may result in participants that have higher education levels and a higher proportion of women than the targeted population of people experiencing grief symptoms after bereavement or romantic separation [79]. However, the study will provide valuable information about the efficacy of LIVIA 2.0 for the targeted population. Second, because participants will have considerable freedom in the way they navigate through the program (ie, choosing the order of the modules and selecting the exercises they want to complete in each session), all the participants will not have taken the same path to complete LIVIA 2.0. However, this will provide them with more choices and higher individualization. Finally, we cannot confirm accurately if the participants respect the exclusion criteria of not undergoing face-to-face psychotherapy while doing the program. This would not be ethically nor practically feasible. However, as the participants are randomized to both programs, this potential bias should be equal in both study arms.

### Conclusions

IBIs contribute to closing the so-called treatment gap, which refers to the difference between the number of people needing psychological treatment and those who actually undergo it [80,81]. This is particularly true in the case of interventions targeting grief symptoms, as a significant proportion of people reporting the need for support after bereavement do not get it [82,83]. Additionally, psychotherapists often lack the competences to deal optimally with this affection [84,85]. Finally, the COVID-19 pandemic crisis has contributed to the increased risk of suffering complicated grief symptoms [86,87]. Hence, developing, assessing, and offering IBIs for grief-related symptoms seems particularly crucial in the current times [78,86]. The results of this RCT will give insight into the relevance of the present developments in outcome improvement and dropout diminution for adults who experience grief symptoms. Besides, the study design will allow for conducting additional analyses that can provide a deeper and more fine-grained understanding of the mechanisms of change in IBIs. For example, we will be able to analyze the effect of specific modules on the weekly mood and symptom monitoring, assess the effect of some moderators (eg, attachment style or closeness to the lost person at pretest), or study the linguistic behaviors in the exercises where participants are required to describe a loss-related situation [39].

## Appendix

Multimedia Appendix 1 Calendar of the participants’ measures.

Multimedia Appendix 2 Feedbacks from Fonds National Suisse Schweizerischer Nationalfonds (SNSF, Swiss National Science Foundation) reviewers.

## Abbreviations

DPM: Dual Process Model
IBI: internet-based intervention
MOTR: Motive-Oriented Therapeutic Relationship
RCT: randomized controlled trial
REDCap: Research Electronic Data Capture

## Data Availability

The data sets that will be generated during the current study will be available in the Zenodo repository. They will be password-protected to ensure correct use of the data, but the data will be made available from the corresponding author upon reasonable request.
