# Supplementary material for: Supporting People Who Have Lost a Close Person by Bereavement or Separation: Protocol of a Randomized Controlled Trial Comparing Two French-Language Internet-Based Interventions
Source: JMIR Res Protoc. 2022 Jun 23;11(6):e39026. doi: 10.2196/39026 (PMC9264124; doi:10.2196/39026)
Supplement: Multimedia Appendix 1 [file resprot_v11i6e39026_app1.pdf]

Appendix 1: Calendar of the participants' measures

**Visits**	**Screening**	**Enrolment**	**Pre-test**	**Intervention**	**Monitoring**	**Monitoring SAE**	**Post-test**	**Follow-up**
Description	Online (Redcap)	Online (Redcap) + e-mail	Online (Redcap)	Online (Programmes LIVIA 1 + LIVIA 2.0)	Online (Programme LIVIA 2.0)	Online (Programme LIVIA 1 + LIVIA 2.0)	Online (Redcap)	Online (Redcap)
Days	*Day -3*	*Day -3*	*Day 0*	*Days 0-90*	*Days 1, 15, 29, 43, 57, 71, 85*	*Days 8, 22, 36, 50, 64, 78*	*Day 90*	*Day 180*
Eligibility	X							
The suicidal ideation attributes scale (SIDAS-FR)	X							
Signature consent (pdf)		X						
**Primary outcomes**								
The Traumatic Grief Inventory Self Report (TGI-SR)			X				X	X
The Patient Health Questionnaire (PHQ)-9			X				X	X
Flourishing scale (FS)			X				X	X
**Secondary outcomes**								
Generalized Anxiety Disorder (GAD-7)			X				X	X
Coping with bereavement questionnaire (QCD)			X				X	X
Self-concept clarity scale (SCC)			X				X	X
Centrality of Event Scale (CES)			X				X	X
Index of self-continuity			X				X	X
UCLA Loneliness Scale			X				X	X
Mood					X			
Grief symptoms					X			
LIVIA satisfaction scale							X	
**Moderators:**								
Demographic data		X						

Experiences in Close Relationships Scale (ECRS)			X					
Couple satisfaction (DAS-IV)			X					
The Inclusion of the Other in the Self Scale (IOS)			X					
**Security outcome**								
Question about serious Adverse Event (SAE)						X	X	X
**Descriptive variables**								
Data entered by the participant				X				
Data entered by the program				X				
