# Supplementary material for: Supporting People Who Have Lost a Close Person by Bereavement or Separation: Protocol of a Randomized Controlled Trial Comparing Two French-Language Internet-Based Interventions
Source: JMIR Res Protoc. 2022 Jun 23;11(6):e39026. doi: 10.2196/39026 (PMC9264124; doi:10.2196/39026)
Supplement: Multimedia Appendix 2 [file resprot_v11i6e39026_app2.pdf]

# Review: 3

## Application data

**Applicant(s)**

Debrot, Anik

Pomini, Valentino

**LIVIA-FR: An online unguided self-help intervention for people struggling with interpersonal loss**

Project funding in humanities and social sciences (Division I)

## Detailed evaluation

### Applicants' scientific track record and expertise

Although the applicant is a junior investigator, she and her team appear well qualified to conduct this study.

### Scientific relevance, originality and topicality

The application is quite novel in several respects -- testing an unguided online grief intervention relative to a guided intervention and focusing on non-death losses.  There is also a need for online grief resources in French.

### Suitability of methods and feasibility

The methods appear to be sound and reasonable for the most part. There are some questions/reactions this reviewer had to the study design and these comments are:

1) They are recruiting help-seeking bereaved adults.  They may have trouble demonstrating significant effects if the sample does not, for example, meet criteria for Prolonged Grief Disorder or some threshold for "caseness", as there will be little room for improvement
2) While the feature of allowing participants to choose their own way is admirable, given the tendency for people to avoid the thing that is hardest for them (eg, reviewing painful experiences), there may be a tendency to work on areas that are less problematic so there should probably be a check on this
3) the sample size estimates are reasonable, but they may well have trouble identifying 234 participants within the time frame of the study. can they address how they may take steps to ensure proposed sample targets are met?
4) 12 weeks is a long time and many may wish for it to be shorter and shorter duration would probably limit substantial attrition
5) the aspect of social touch is interesting but it was not that obvious why the intervention would alter someone's receptivity to touch. would be helpful to connect those "dots" more explicitly
6) it is unclear why they are not using validated criteria for Prolonged Grief Disorder and/or the PG-13

which is the "best of" the ICG

**Comment**

This is a very much needed, innovative and sound study that is likely to benefit bereaved individuals with prolonged grief.  It is innovative and addresses some aspects of online bereavement interventions that need to be tested -- explicitly the necessity of the online grief "guide".  Looking forward to seeing this work published in the future and learning from it.

## Note on the evaluation procedure

The evaluation bodies of the SNSF strive to reach a balanced overall assessment of each proposal. External reviews play an important role in this. Reviewers generally review only one proposal. The evaluation bodies of the SNSF, however, must compare and rate the quality of all proposals submitted by a given deadline. The opinions expressed in external reviews are generally positive, or they may occasionally include critical remarks that are largely irrelevant to the assessment conducted by the evaluation body. Therefore, the final decision taken by the SNSF evaluation bodies need not necessarily reflect the content of external reviews.

## Review: 2

### Application data

**Applicant(s)**
Debrot, Anik
Pomini, Valentino

**LIVIA-FR: An online unguided self-help intervention for people struggling with interpersonal loss**
Project funding in humanities and social sciences (Division I)

### Detailed evaluation

### Applicants' scientific track record and expertise

Anik Debrot, PhD
Dr. Debrot is published in high quality peer-reviewed journals and has sufficient research expertise to carry out the project.

Valentino Pomini, PhD
Dr. Pomini's also has a good record of publication. I am not familiar with many of the journals Dr. Pomini has published in because they are French speaking, but this work appears to indicate sufficient research expertise to carry out the project. However, Dr. Pomini does not appear to have any first-authored empirical publications.

The research team possesses both methodological and clinical expertise that is certainly sufficient for the project. However, neither of the applicants has significant expertise or domain knowledge in grief. This is an important concern and is reflected in their reliance on Worden's work, which has not been validated empirically and has exerted little influence on recent theoretical developments in grief (e.g., Bonanno's work on resilience and tests of grief work models; MaCallum's recent conceptual work on grief).

### Scientific relevance, originality and topicality

The project has scientific relevance, addresses an important social problem, and takes advantage of a delivery system with broad accessibility and potential for dissemination. I certainly think there is value in comparing a guided and an unguided IBI intervention. The authors display a good understanding of the grief literature, but they neglect a number of thinkers and ideas on grief with clinical relevance. For example, current CBT approaches emphasize exposure, both imaginal and situation, but there is little discussion of these approaches. Instead, the applicants rely on Worden's work, which has little empirical support.

The project is incremental in focus but contains some original components. The most original components are the introduction of touch as a therapeutic component, the use of an unguided intervention, and the introduction of a tailored approach that allows participants to select the

intervention components they are interested in. The project is certainly topical and pertains to a substantial scientific literature on treatment approaches for grief.

**Suitability of methods and feasibility**

The project is certainly feasible and in the broad sense uses suitable methods. However, I had a number of concerns about the research design and some components of the intervention itself. These concerns are related.

My primary concern is that the design does not permit an evaluation of the intervention being proposed. There are two reasons for this. The first is that the LIVIA-FR-I and II differ not just in being unguided but in having different intervention components. In effect, they have a 2 (guided vs. unguided) x 2 design (intervention I and intervention II). But their design is one-way. If the proposed intervention is superior to the LIIA-FR-I, it will not be possible to determine whether that is attributable to the unguided component or the intervention component. Equally important, if the intervention performs more poorly, the researchers will not know why. I would think it preferable to use the same intervention in guided and unguided formats.

The second reason is that the IBI intervention does not appear to have a published empirical article demonstrating its efficacy. If the LIVIA-FR-I is efficacious, the applicantsshould discuss in greater the evidence for that efficacy in theirproposal. If it has not been fully evaluated, the applicants shouldintroduce a control arm to the design, likely a wait list control or anull intervention. As it is, the present design will not allow the researchers to detect the efficacy of either version of the intervention. But a control arm would allow them to test each intervention against no treatment. This would also begin to create the conditions for a non-inferiority trial (comparing a less intensive intervention with a more intensive intervention one to see if they produce equivalent results). It should be noted that a non-inferiority trial involves accepting the null. But this is only possible when one of the interventions has demonstrated efficacy. It should also be noted that the power calculation of a small effect would not apply to that comparison. (I would also note that the power calculation was confusing to me. I would not expect an unguided intervention to produce superior results to a guided intervention. Instead the hope would be that they would produce equivalent results.)

Another concern is that the intervention rearranges the LIVIA-FR-I and introduces some new intervention components. It was not clear to me that these changes were empirically justified. A particular concern is that the participants are allowed to choose their own intervention components. It is an empirical question as to whether this preferable. I have treated people suffering from prolonged grief using exposure treatments. It was clear to me that they would often do anything to avoid the exposure component of the treatment. However, that component was the most important and efficacious one, as the data show. Allowing participants to opt out of the exposure component of this intervention, which would correspond to module 5, would potentially result in a less efficacious treatment.

**Comment**

The primary strengths:  1) the development of an unguided IBI is a clear strength of the proposal. 2) the intervention components included in the unguided IBI are largely consistent with current intervention approaches with one exception (the absence of a situational exposure component), and this is also a strength. 3) The capacity to widely disseminate the intervention is a clear strength.

But these strengths are qualified by some important weaknesses. Indeed, in order for the strengths to be realized, the weaknesses must be addressed in my view. The primary weaknesses are: 1) an excessive reliance on a model of grief (Worden's tasks of grieving) without clear empirical support; 2) a research design that will not permit a clear inference regarding what components of the intervention were efficacious (or not efficacious); 3) .

**Note on the evaluation procedure**

The evaluation bodies of the SNSF strive to reach a balanced overall assessment of each proposal. External reviews play an important role in this. Reviewers generally review only one proposal. The evaluation bodies of the SNSF, however, must compare and rate the quality of all proposals submitted by a given deadline. The opinions expressed in external reviews are generally positive, or they may occasionally include critical remarks that are largely irrelevant to the assessment conducted by the evaluation body. Therefore, the final decision taken by the SNSF evaluation bodies need not necessarily reflect the content of external reviews.

## Review: 1

### Application data

**Applicant(s)**
Debrot, Anik
Pomini, Valentino

**LIVIA-FR: An online unguided self-help intervention for people struggling with interpersonal loss**
Project funding in humanities and social sciences (Division I)

### Detailed evaluation

### Applicants' scientific track record and expertise

Anik Debrot is the responsible applicant. She is lecturer at the University of Lausanne. She has conducted research on facets of affectionate and non-verbal contact in couple relationships. Her research outputs essentially deal with this theme of interest. Recently, she has become a certified psychotherapist and has translated the guided internet based intervention (IBI) programme LIVIA constructed by Brodbeck, Berger, & Znoj (2017) which helps bereaved persons dealing with prolonged grief reactions. She has thus developed a collaboration with Brodbeck and Hans Znoj who is a wellknown researcher in the field of grief and bereavement and particularly on the validation of IBI for bereaved and divorced people. Her scientific record is good and I believe she has the relevant qualities to conduct the proposed research project.

Valentino Pomini is the other applicant. He is full professor at the University of Lausanne. His research interests deal with the development of self-assessment tools to measure patients' difficulties and needs (ELADEB) and strenghts and resources (AERES). A second area of interest deals with research on psychotherapy and training programmes to help therapists repair therapeutic ruptures. Although these do not directly deal with IBI, it is clear that he has the research qualifications to support this new project. His research record is good and has been invited for lectures and organised symposia in France and Switzerland and contributed to quite a lot of oral presentations during the last 5 years, and participated in editorial work. He also contributed to the translation of the Brodbeck et al. IBI programme into French.

Jeannette Brodbeck and Hansjörg Znoj who are partners of the project co-developed the IBI that will be the control treatment in the project (in an adapted unguided version). They will be valuable ressources for the implementation of the project. Finally, Tanja Bellier-Teichmann is also a partner. She developed the AERES and this instrument will be adapted to an online version for this project.

### Scientific relevance, originality and topicality

The research project is very well described, argued, making recent and relevant literature reviews on grief processes (main models), Internet based interventions, especially those addressing loss-of-relationship ones (bereaved and/or divorced people) and the relevance to test unguided (less human ressources) versions of such IBI is well demonstrated, this consisting in the originality of the project. This is an excellent research project that might have important applications to improve the well-being or prevent detrimental illhealth effects of bereavement and/or divorce on people.

## Suitability of methods and feasibility

The proposed project will use a RCT comparing two unguided IBI, one who will be an adapted unguided version of a programme already developed by Brodbeck et al. and the other who will be developed according to current wellknown theories of adjustment to bereavement/grief (Worden + Stroebe & Schut). A pre-post and follow-up design will be used with various dependent variables assessing the relative impact of the programmes. The two IBI are very well described and convincing.

Although the reason for not including a control group was well-argued, I still wondered whether it should not be the case since the efficacy of both IBI is still not demonstrated. Previous research on unguided IBI, at least one of the studies reported with bereaved people, did not show positive results on grief symptoms, which is the first/primary expected outcome of interest (Van der Houwen et al., 2010). One can thus wonder whether such result will be shown in the present research.

I was also unsure in the description of the project whether there would be measures of adherence to the treatments (I suppose so but from my reading of the project this is not explicitly stated). These checks should be included since it is an important part of the explained variance of the efficacy of IBI. It should be stated that the applicants have tried to maximize adherence with different automatic strategies.

With regard to feasibility, the plan is well described and realistic.

## Comment

Both applicants have very good scientific records and skills that make, in collaboration with their project partners, the project very original, relevant for theoretical, empirical and clinical reasons. The proposed method is adequate and will provide interesting results.

## Note on the evaluation procedure

The evaluation bodies of the SNSF strive to reach a balanced overall assessment of each proposal. External reviews play an important role in this. Reviewers generally review only one proposal. The evaluation bodies of the SNSF, however, must compare and rate the quality of all proposals submitted by a given deadline. The opinions expressed in external reviews are generally positive, or they may occasionally include critical remarks that are largely irrelevant to the assessment conducted by the evaluation body. Therefore, the final decision taken by the SNSF evaluation bodies need not necessarily reflect the content of external reviews.
